# Biogeographical zonation of rocky intertidal communities along the coast of Peru (3.5–13.5° S Southeast Pacific)

**DOI:** 10.1371/journal.pone.0208244

**Published:** 2018-11-30

**Authors:** Bruno Ibanez-Erquiaga, Aldo S. Pacheco, Marcelo M. Rivadeneira, Claudia L. Tejada

**Affiliations:** 1 Laboratorio de Ciencias del Mar, Universidad Peruana Cayetano Heredia, Lima, Perú; 2 Asociación CONSERVACCION, Lima, Perú; 3 CENSOR Laboratory, Instituto de Ciencias Naturales Alexander von Humboldt, Universidad de Antofagasta, Antofagasta, Chile; 4 Laboratorio de Paleobiología, Centro de Estudios Avanzados en Zonas Áridas, Coquimbo, Chile; 5 Departamento de Biología Marina, Facultad de Ciencias Biológicas, Universidad Católica del Norte, Coquimbo, Chile; 6 Departamento de Biología, Universidad de La Serena, La Serena, Chile; Universita degli Studi di Genova, ITALY

## Abstract

The biogeography of the Peruvian Eastern Pacific coast has been described based on oceanographic parameters and qualitative species occurrence data. This has generated disagreement about the limits and existence of different biogeographic units. In this study, the distribution of rocky-shore macrobenthic communities were recorded over 41 sites along the Peruvian coastline (3.5°S-13.5°S) and analyzed together with historic abiotic data in order to quantitatively evaluate the biogeographic zonation of rocky intertidal communities throughout the region and its relationship with environmental variables to propose an update bioregionalization. Clusters and non-metric multidimensional scaling were performed using Bray-Curtis dissimilarity matrices from abundance data to evaluate biogeographic patterns of dissimilarities of rocky-shore communities. Significant turnover of taxa among defined biogeographical units was tested using permutational multivariate dispersion. Relationships between of the biogeographical community’s structure and environmental factors were examined using Random Forest analysis on datasets available at Bio-Oracle and Jet Propulsion Laboratory—California Institute of Technology. Variation of community structure of 239 taxa depicted three biogeographical units along the region matching Panamic, transitional and Humboldt provinces. Beta diversity analysis indicated a significant turnover of taxa within the transitional unit. Random forest analysis showed a strong correlation between biogeographic units with phosphate, sea surface temperature, nitrate, dissolved oxygen, cloud fraction, and silicates. Our results set the putative limits of three biogeographic units for rocky-shore communities along the coast of Peru, providing base-line information for understanding further biogeographic changes on communities associated with the ongoing regional coastal cooling and impacts of El Niño events.

## Introduction

Understanding the spatial patterns of biodiversity along latitudinal gradients is a major task in coastal biogeography [1, 2]. The biogeographic classification of nature builds a better understanding for conservation strategies and resource management [3, 4]. Although such endeavors started several centuries ago [5, 6], the ongoing transformation of marine regions by anthropogenic factors, in synergy with climate change, calls for a renewed and updated research on biogeographical spatial patterns [7–9].

Biogeographical zonation of coastal regions has used single species distribution ranges [10, 11], endemism [12, 13] and associations between taxa and oceanographic variables from existing databases to build broad-scale classification [14, 15]. The development of multivariate statistical techniques and better access to comprehensive abiotic datasets allows a more integrative description of biogeographic patterns [3, 16]. Early studies on biographic zonation of rocky intertidal organisms focused on single taxa patterns [10, 17, 18], whereas community level (i.e. species weighted per their relative abundance) research has received more attention in recent years [2, 19, 20]. Assessing the variation of communities may capture patterns that cannot be revealed by single taxa analysis. Blanchette et al. [20] analysed rocky intertidal communities of the Pacific coast of North America and while they corroborate previous descriptions of biogeographic units, they demonstrated regional variations in community patterns. In South Africa, Sink et al. [21] found several species co-occurring in both tropical Maputaland and subtropical Natal rocky intertidal despite the distinct affinities of these biogeographic units, which were only revealed through examination of community abundance variation. In rocky intertidal areas of the west coast of North America, sub-set of species would be present along the gradient but with remarkably different abundances associated with distinct temperature values [22]. Arguably, analysis of rocky intertidal communities seems to be sharper than single taxa in delimiting biogeographic zonation since this level may greater capture environmental variation (e.g. wave stress and temperature gradients) and ecological interactions (e.g. invasion, predation and space competition), providing an improved resolution of spatial patterns [23–25]. The variation in beta diversity (turnover in taxa composition throughout a gradient [26]) could serve to complement community structure analyses due to compositional changes of the community between areas with contrasting environmental characteristics that act as filters [27, 28]. Such filtering may occur at breaks and transitional zones at biogeographical boundaries [29, 30].

The coastline of Peru spans ca. 3080 km from 3.5 to 18° S and represents 19% of South American west coast (https://www.cia.gov/library/publications/the-world-factbook/). Most of this coastal region is characterized by wind-induced upwelling flowing northward reaching the equator [31, 32]. Hence, the region has characteristically cold waters in comparison to other continental margins at the same latitude, thus resembling temperate conditions at tropical latitudes [31, 33]. This region is subject to inter-annual variability associated with the intrusion of Kelvin waves bringing warm equatorial and oceanic waters to the coast during strong El Niño events [33, 34]. During El Niño, range extension of several species distributed primarily at the equatorial region into latitudes where normally cold-upwelling species occur [33, 35, 36]. Even though such distributional changes in species may intuitively imply changes in the extent of biogeographic units, such variability remains poorly understood. From the biogeographic point of view, the region represents a challenge in terms of predicting biotic spatial distributions in the context of anthropogenic climate change. Most coastal areas worldwide are warming [37, 38], while the coasts of central Peru and northern-central Chile have had an ongoing cooling trend since ca. 1950 [37, 39, 40]. Accordingly, it has been shown that species range shifts could be distinct of poleward directed following the heterogeneous nature of temperature change worldwide [38, 41, 42]. On the other hand, strong El Niño events are predicted to increase in frequency because of global change [43, 44]. In this scenario, there is an urgent need in this region for biogeographical studies involving systematic surveys along a wide latitudinal gradient to help us understand the spatial distribution of coastal communities.

Two biogeographic provinces have been described for the coast of Peru: the Panamic Province (Tropical East Pacific), correlated to the Ecuador-Peru Coastal Current; and the Peruvian Province (Warm Temperate Southeastern Pacific), associated with the upwelling Humboldt current [15, 45, 46]. While the existence of an ecotone (i.e. transitional zone) between these units at ca. 4° S has been proposed, its extent and limits are not well defined [46–48]. Variation on the extent of such limits could be attributed to the fact that some studies have assumed species ranges or analyzed databases with confounded temporal shifts in spatial distribution patterns during El Niño years rather than verified sampling data [49]. The biogeographical zonation of the Peruvian coastline has been primarily based on oceanographic surrogates [50, 51], single taxa [18] and data revision combined with specialist prior knowledge [15]. So far, no studies have specifically surveyed communities to describe the biogeographical zonation. Herein, we tested the prediction of three biogeographic units including the Panamic and Peruvian provinces with a transitional zone in between. Additionally, we investigated the abiotic variables influencing the observed patterns of community structure.

## Methods

### Study localities and sampling sites

**Fig 1:**
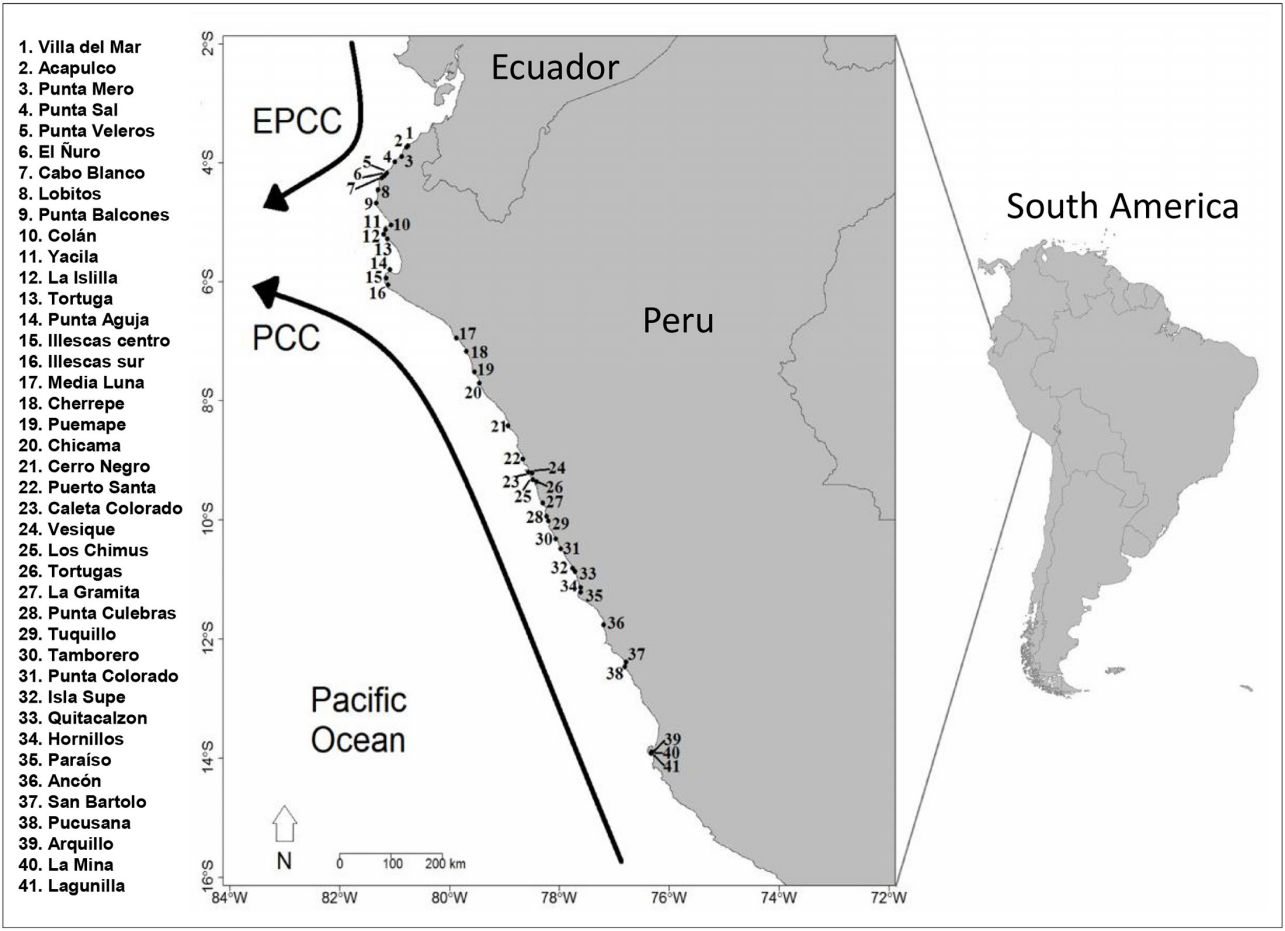
Map of the study area covering the coast of Peru. Dots with numbers represent sampling sites. Solid arrows represent the two main currents: Ecuador-Peru Coastal Current EPCC and Peru Coastal Current PCC [45].

21 localities (i.e. groupings of neighboring sites) were sampled throughout the coast of Peru, from 3.5 to 13.5° S, keeping 0.5° of latitudinal distance between localities. In each locality, one to three sites (i.e. beaches with intertidal rocky platforms) were sampled. The number of sites and the distances between them varied depending on the presence and extension of sandy beaches. A total of 41 sites were sampled (Fig 1). This approach allows evaluating latitudinal patterns of intertidal community structure [52, 53]. Overall, each sampling site consisted of gently sloping rocky platforms (<45° angle), with tide height variation range of 1 to 2.5 m, similar morphology (tide pools or crevices were excluded), sheltered from wave exposure and as far from human settlements as possible or any source of evident pollution. These criteria were used to minimize between-site heterogeneity associated with physical conditions. Sampling was conducted at the end of the austral summer and beginning of autumn (wet season) between February and May 2015. This timing did not encompass a strong seasonal change. The sampling period was categorized as “neutral to moderate” conditions of the El Niño-Southern Oscillation (ENSO) cycle according to Comite Multisectorial Encargado del Estudio Nacional del Fenomeno El Niño (http://senamhi.gob.pe/?p=0805).

### Sampling strategy

At each sampling site, four transects parallel to the coastline were deployed at high (one transect), middle (two transects) and low (one transect) rocky intertidal. Intertidal levels were identified based on the presence of indicator species of each zone; high (litorininds), middle (barnacles and mussels) and low (large macroalgae) [54]. Four quadrats of 0.5 by 0.5 m (0.25 m^2^ area, gridded with 100 intersection points) were randomly placed throughout each transect. A total 16 quadrats per site spanning the area, placed at varying distances between them but up to a maximum shore band of 50 m, were used to quantify the community. This sampling effort has been previously reported to be sufficient to represent the structure of rocky communities along the Southeast Pacific coast [52]. The abundance of motile invertebrates was estimated by counting individuals directly within the surface area delimited by the quadrat. Percentage cover of macroalgae and sessile invertebrates was estimated by counting the intersection points of the grid. Abundant small mobile invertebrates (e.g. litorinids) were sub-sampled in a 0.0625 m^2^ area of the quadrat. Organisms under algae fronds were also quantified, and sessile species overlapping each other were estimated (i.e. total percentage could exceed 100% within a quadrat). Only organisms visualized with naked eye were quantified. Invertebrates and macroalgae were usually identified at species level *in situ*, except for cryptic taxa. Unidentified samples were taken for further laboratory examination. Sampling always occurred during daylight and at the lowest tide hours; this information was provided by Dirección de Hidrografía y Navegación—Marina de Guerra del Peru (https://www.dhn.mil.pe/mareas). No humans or vertebrate animals were used in this study and the methodology used was accepted by the animal ethics committee at Universidad Peruana Cayetano Heredia (project code 64776). Field permit for sampling at Illescas was granted by Servicio Nacional de Áreas Naturales Protegidas por el Estado.

### Environmental variables

For each locality, we obtained information of environmental conditions based on annual climatologies (of variable length) of 20 variables; these are shown in S1 Table. Sea surface temperature was obtained from the database of the Jet Propulsion Laboratory at the California Institute of Technology (https://mur.jpl.nasa.gov/), which provides 12-year mean at 1-km resolution values 5 km offshore of each site. The other variables were taken from Bio-ORACLE, an environmental database for marine spatial analysis [55], which has been used by previous biogeographic studies as proxies of upwelling conditions (e.g. [56]), and correspond to satellite data combined with *in situ* measurements, and then interpolated in global grids with 5 arcmin (~9.2 km). Each sampling site was matched to the closest available variable data.

### Data analysis

To visually explore patterns of biogeographical zonation at the study region, non-metric multidimensional scaling (NMDS) ordination plots were constructed from the Bray-Curtis dissimilarity matrix using square-root transformation of the data to counterbalance the contribution of rare and very abundant species to the dissimilarity. NMDS ordination plots were built for matrices based on abundance and cover percentage of taxa respectively. In addition, for the examination of the biogeographical patterns of the whole community structure, the routine “combined MDS” [57] was used to produce a single NMDS that captures all the information in the samples (counts and cover percentage together). This routine estimates the average of the best positions of the two Sheppard diagram (from each NMDS) to build one combined ordination. For these analyses, average data every 0.5° latitude was used. Cluster analysis was conducted to examine the variation in similarity every 0.5° latitude. Clusters were constructed from the Bray-Curtis dissimilarity matrix calculated using the presence/absence data, thus removing the effect of estimations coming from different measures (percentage cover versus number of individuals). These analyses outputs were superimposed (cluster over the combined MDS) to depict latitudinal grouping. Furthermore, group-average linking was used to build similarity dendograms and the similarity profile analysis (SIMPROF) was used to detect significant grouping at *P* = 0.05 [58]. Since the complete data comprised 239 taxa (see Results section), the BVSTEP routine was used on the abundance and percentage of cover matrices to detect the subset of taxa which generates the same multivariate pattern as that obtained from the entire community set [57]. This analysis uses Spearman rank correlation to determine the minimum number of taxa that show the highest correlation with the complete dataset. To examine the taxa turnover along the latitudinal gradient (beta diversity), a test of homogeneity of multivariate dispersion (PERMDISP) was conducted from the similarity matrix calculated from the Sørensen index using taxa composition from presence records [59]. Multivariate analyses were conducted using PRIMER 7 [57]. We used Random Forest analysis [60, 61] to relate the biogeographic structure with the environmental variables. Random Forest is a powerful machine-learning method of growing use in ecology and biogeography, and it is based on an assemblage of bootstrapped classification trees [62, 63, 56]. This non-parametric method deals with continuous and categorical responses and makes no assumptions about the residuals of the models, as traditional multivariate regressions. Analyses were carried out using the library “randomForest” [61] in R [64]. The relative importance of each predictor variable was evaluated as the mean decrease in accuracy of the model, and its statistical significance was tested using the permutation algorithm implemented in the library “rfPermute” [65] in R using 50,000 runs. To minimize the effect of multi-collinearity among predictor variables, which could severely bias assessments of variable importance [66], we selected only variables with a correlation threshold of 0.85 using the package “usdm” [67] in R. Only 11 out of 20 variables with reduced multi-collinearity were used in further analyses. Using other collinearity thresholds (0.5, 0.6, and 0.7) slightly reduced the accuracy of the model. The relative importance of each variable was rescaled to take values between zero and one using the expression proposed by Ellis et al. [68], so they can be interpreted as pseudo-R^2^ values.

## Results

### Biogeographic patterns of rocky shore communities

**Fig 2:**
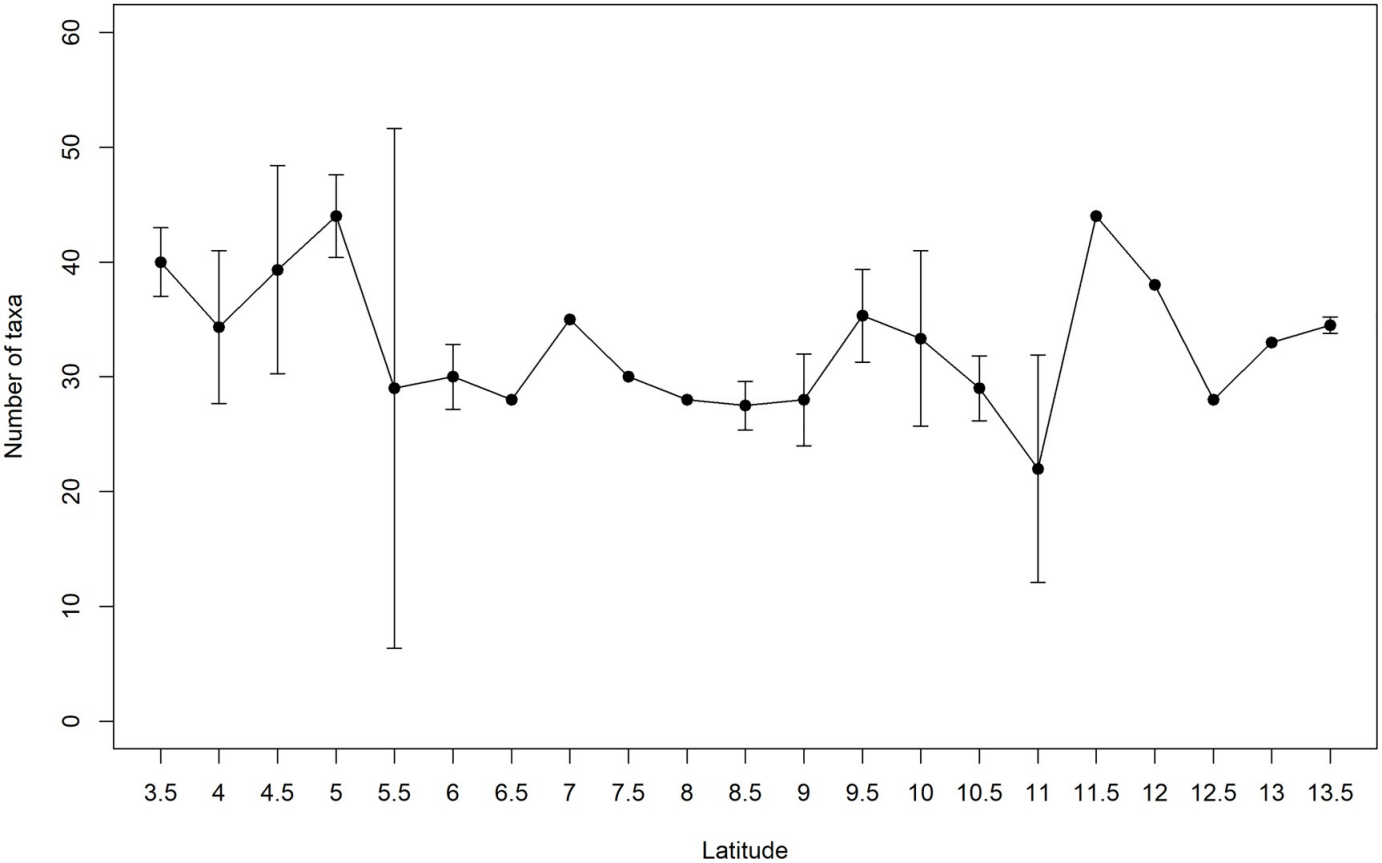
Latitudinal gradient of taxa richness along the study region (3.5–13.5° S). Taxa richness averaged every 0.5 latitudinal degrees. The latitudes without standard deviation had only one site sampled.

**Fig 3:**
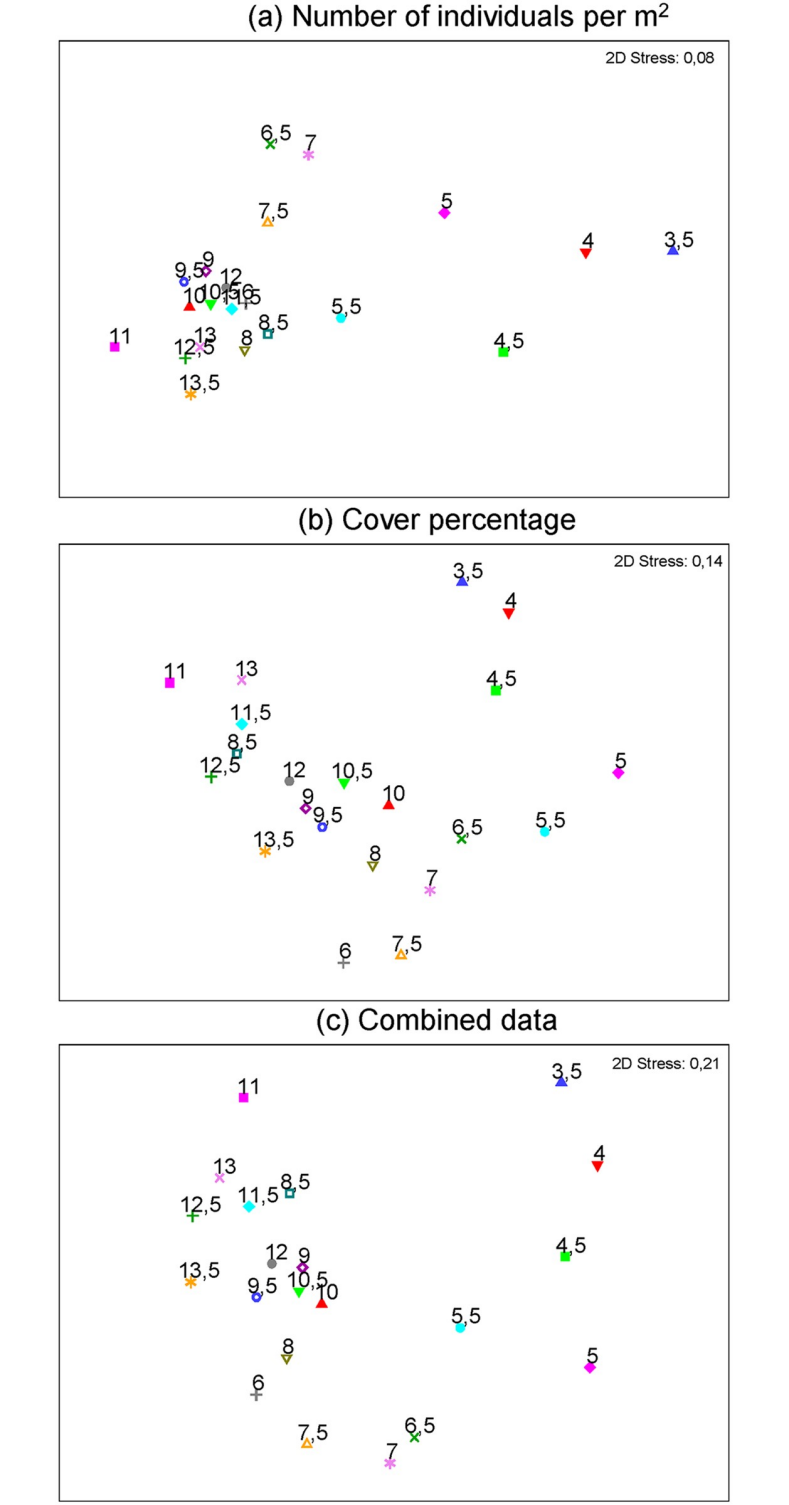
Patterns of community’s latitudinal dissimilarity. NMDS ordination plots of the community structure throughout the study region at 0.5 latitudinal degrees (3.5–13.5 °S) is shown. (a) Dissimilarity pattern for taxa density. (b) Dissimilarity on cover percentage of taxa. (c) Combined nMDS from density and cover data.

**Fig 4:**
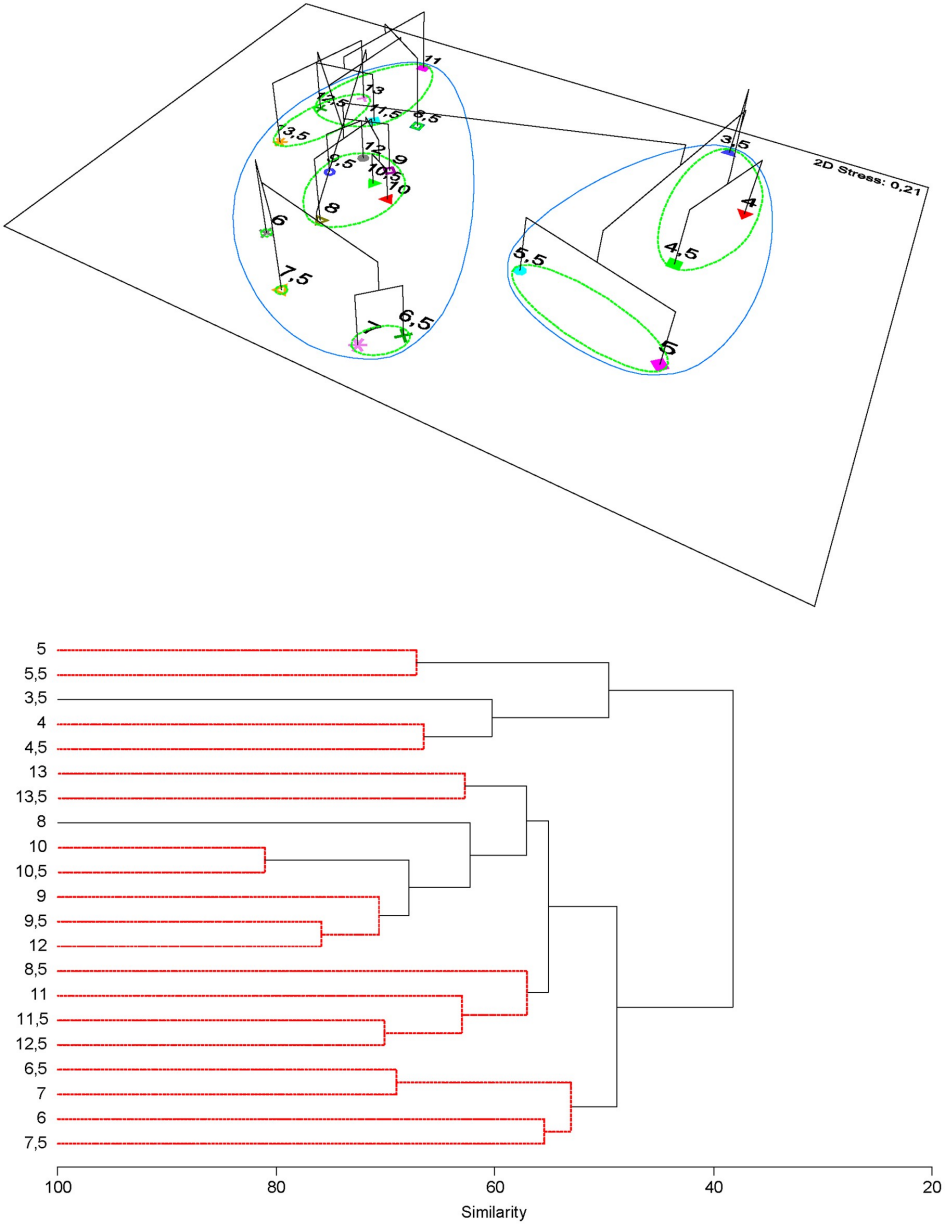
Multivariate depiction of biogeographical units. Upper plot, NMDS ordination with a superimposed dendrogram showing the pattern of biogeographic dissimilarity in community structure (Similar groups at 50% (green) and about 38% (blue) are shown). Below, dendrogram with significant groups (red lines) after SIMPROF test. In both plots the numbers (3.5 to 13.5) represent the localities every 0.5 latitudinal degrees.

We registered 239 taxa which consisted of 154 Mollusks, 53 macroalgae, 12 Cnidaria, 9 Crustacea, 6 Echinodermata, 4 Porifera, and 1 Bryozoa; all detailed data can be found in S2 and S3 Tables. With exception of *Caulerpa* sp. all taxa were native. The localities with highest taxonomic richness were 3.5 and 5° S, with 81 and 80 taxa respectively. The lowest number of taxa was found at 12.5° S with 24 taxa. Rocky intertidal communities along the latitudinal gradient were dominated by macroalgae at sites located between 11.5 and 13.5° S. Overall, higher species richness was found at lower latitudes, higher abundances at southern latitudes and marked variability in between these units (Fig 2). NMDS ordination plots suggested that dissimilarity decreased as latitude increased. Similar localities for abundance, cover percentage, and combined ordinations occurred at 3.5–4.5° S; also at 5, 5.5, 6.5–7.5° S and from 8 to 13.5° S (Fig 3a–3c). Superimposed cluster on NMDS analyses results showed 4 main groups at a 50% similarity: two clear groups within the low latitudes, 3.5–4.5° S, and 5–5.5° S. Towards higher latitudes two groups resulted at 6–7.5° and 8–13.5° S. This ordination generally evidenced a north-south pattern where 3.5–4.5° S represents a northern spatial unit; 5 and 5.5° S belong to an intermediate or transition unit; and 6–13.5° S belong to a southern unit (Fig 4). The cluster analysis with SIMPROF test showed a correspondence with the NMDS results, portraying the independent grouping of northern localities, transitional localities and a southern spatial structure with several groups (Fig 4).

**Fig 5:**
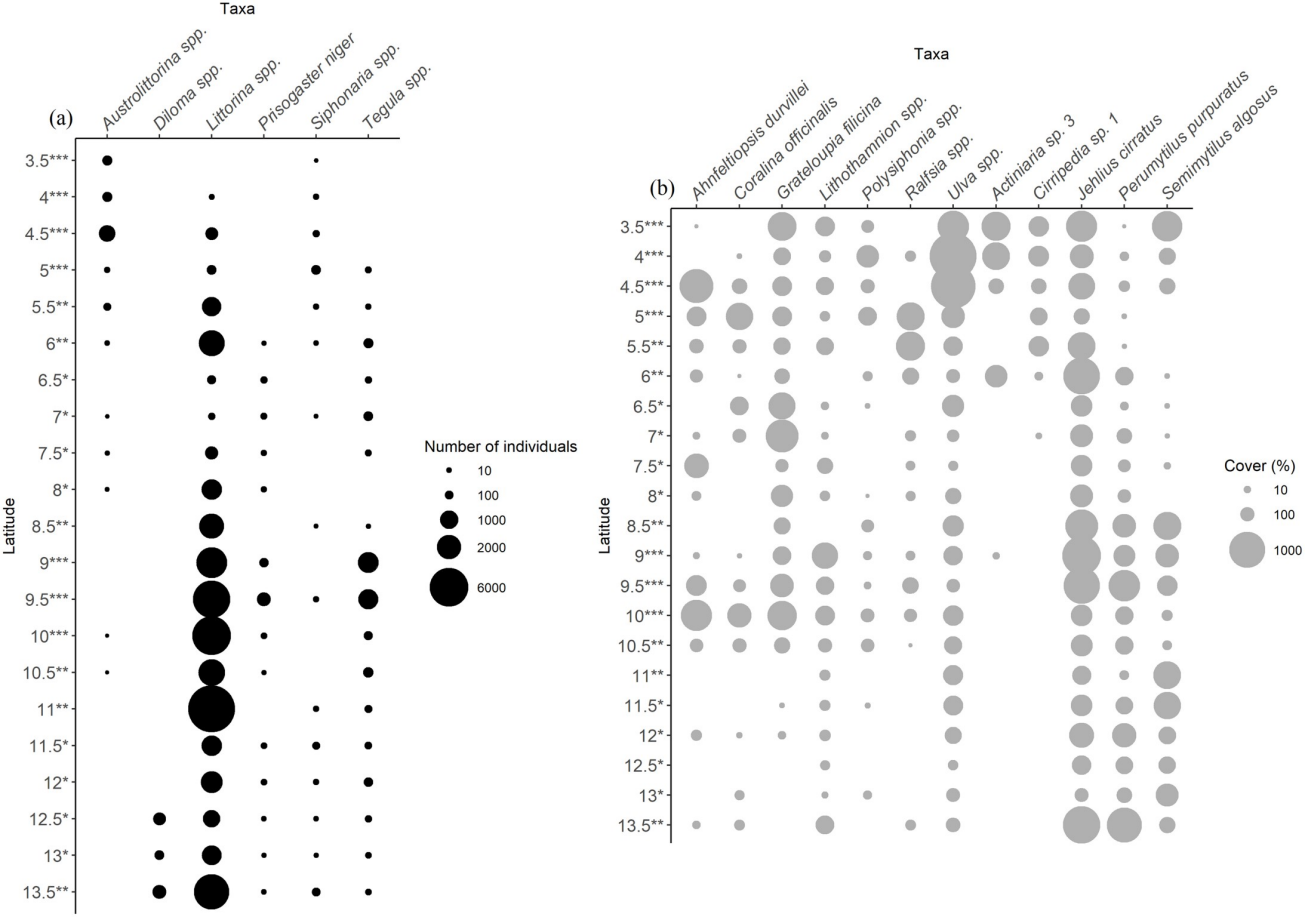
Relative abundance of taxa throughout the latitudinal gradient. Patterns of latitudinal variation on relative abundance of taxa after BVSTEP routine. The data per locality consisting of one (*), two (**) or three (***) sites accumulated comes from (a) number of individuals and (b) percentage cover.

The BVSTEP analyses showed that the pattern of variation in community structure was successfully explained by the following subset of motile taxa: gastropods *Austrolittorina* spp., *Diloma* spp., *Littorina* spp., *Prisogaster niger*, *Siphonaria* spp., and *Tegula* spp. For sessile organisms, the subset of taxa was: macroalgae *Ahnfeltiopsis durvillei*, *Coralina officinalis*, *Grateloupia filicina*, *Lithothamnion* spp., *Polysiphonia* spp., *Ralfsia* spp., *Ulva* spp., the sea anemone *Actiniaria* sp. 3, the barnacle Cirripedia sp. 1, *Jehlius cirratus*, and the mussels *Perumytilus purpuratus* and *Semimytilus algosus*. These subsets of taxa showed correlations of ρ = 0.953 (counts) and ρ = 0.954 (cover) with the complete matrix. The latitudinal variation in abundance of these taxa exhibited the following patterns; higher relative abundance at middle and higher latitudes followed by higher taxa richness at northern localities (Fig 5).

NMDS and cluster analysis suggested that sampling sites constituted three main biogeographic units: Panamic province, a transitional zone and Humboldt (Peruvian) province. Regarding variation of beta diversity, the PERMDISP test detected significant differences among biogeographical units (F = 6.771; df = 2, 606; P < 0.05), but particularly, it identified the transitional zone as significantly distinct from the other two units (P < 0.05 in pair wise comparisons). The average Sørensen distance-to-centroid was higher for the transitional unit (58.083%) than Panamic (51.996%) and Humboldt (53.394%). The difference of 6% between the centroids, although significant, seems to be ecologically important, because the distinction of biogeographical units is also distinguished by random forest analysis (see below).

### Biogeographic zonation and its relationship with abiotic variables

**Fig 6:**
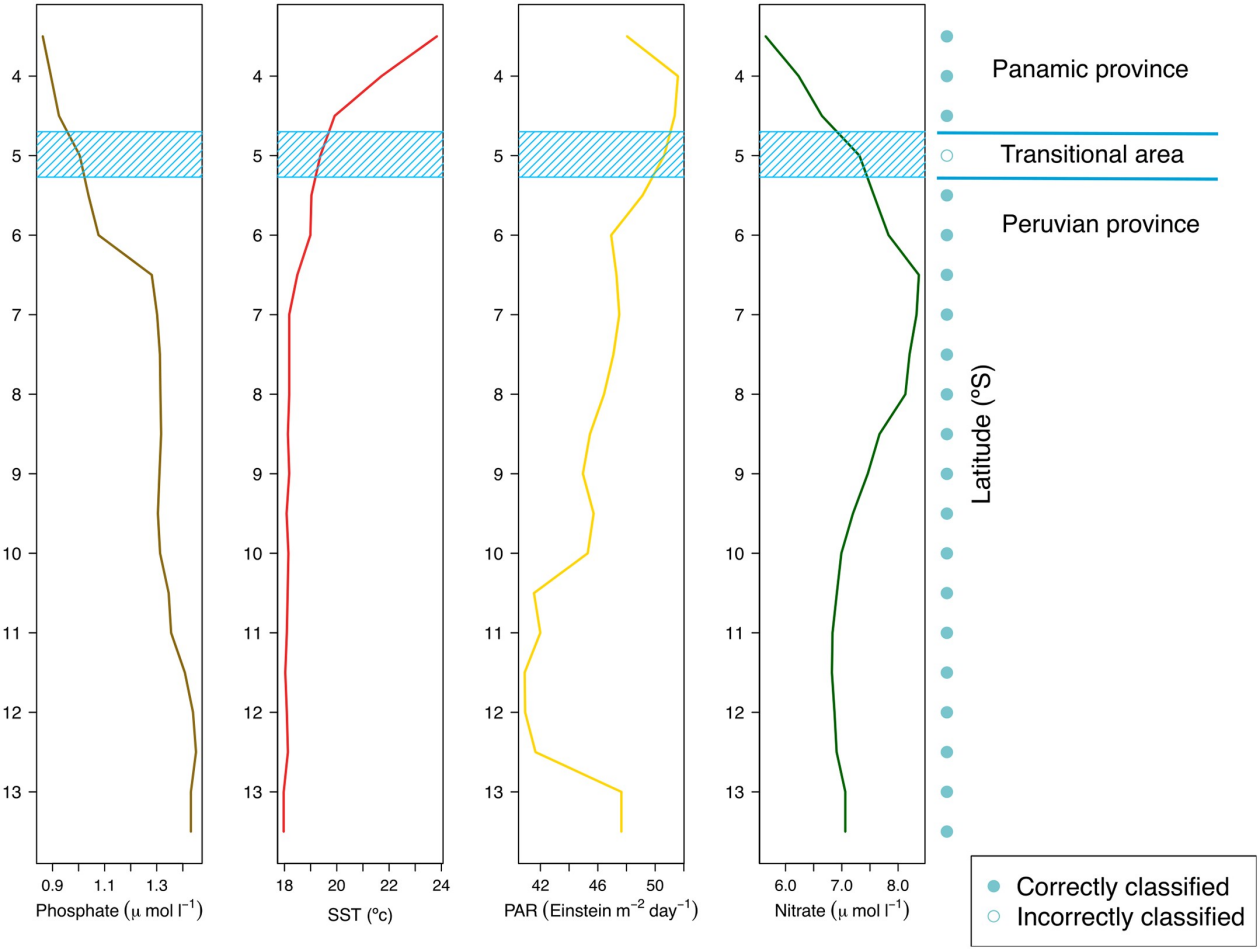
Latitudinal variation of the environmental variables predicting the biogeographic structure. Spatial variation in the four top predictors of the biogeographic structure across the study area. Also shown the accuracy of the Random Forest model to correctly predict the biogeographic unit.

**Fig 7:**
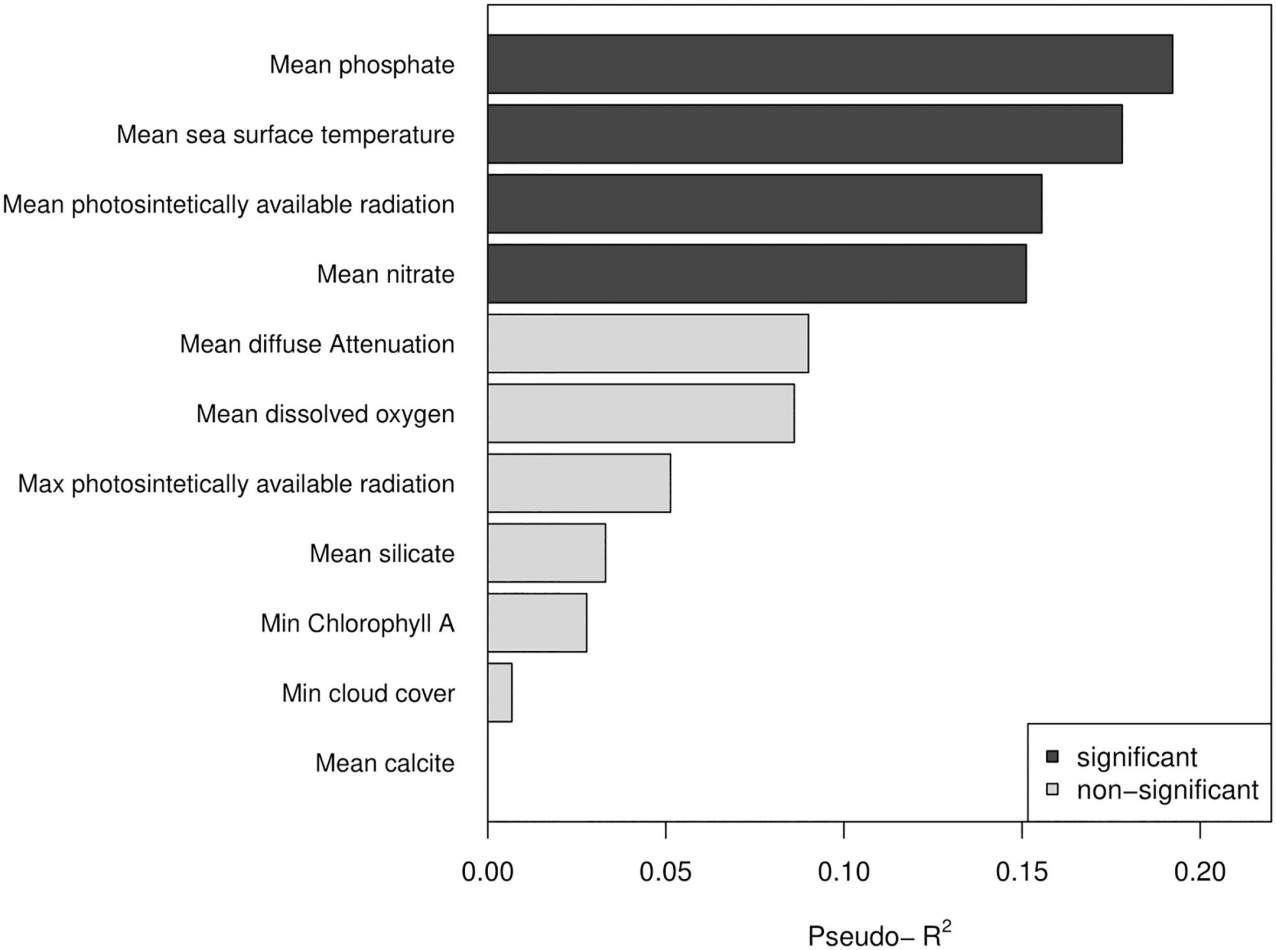
Relative importance of environmental variables predicting the biogeographic structure. Variable importance measured according the pseudo-r^2^ values obtained from the Random Forest model.

The Random Forest model explained the presence of the biogeographic units with a very low error (pseudo-R^2^ = 0.95), i.e. the biogeographic unit assigned to each latitudinal bin was perfectly predicted by the model in 20 out of 21 cases. The only bin that was not predicted by the Random Forest model corresponded to the transitional area (Fig 6). Only 4 out of 11 variables entered to the model were significant (Fig 7); mean phosphate concentration, mean sea surface temperature, mean photosintetically available radiation and mean nitrate concentration. The significant variables showed distinct spatial patterns of variation (Fig 6), but overall, the northern unit was characterized by warmer sea surface temperatures, lower concentrations of phosphate and nitrate, and a higher photosintetically available radiation.

## Discussion

**Fig 8:**
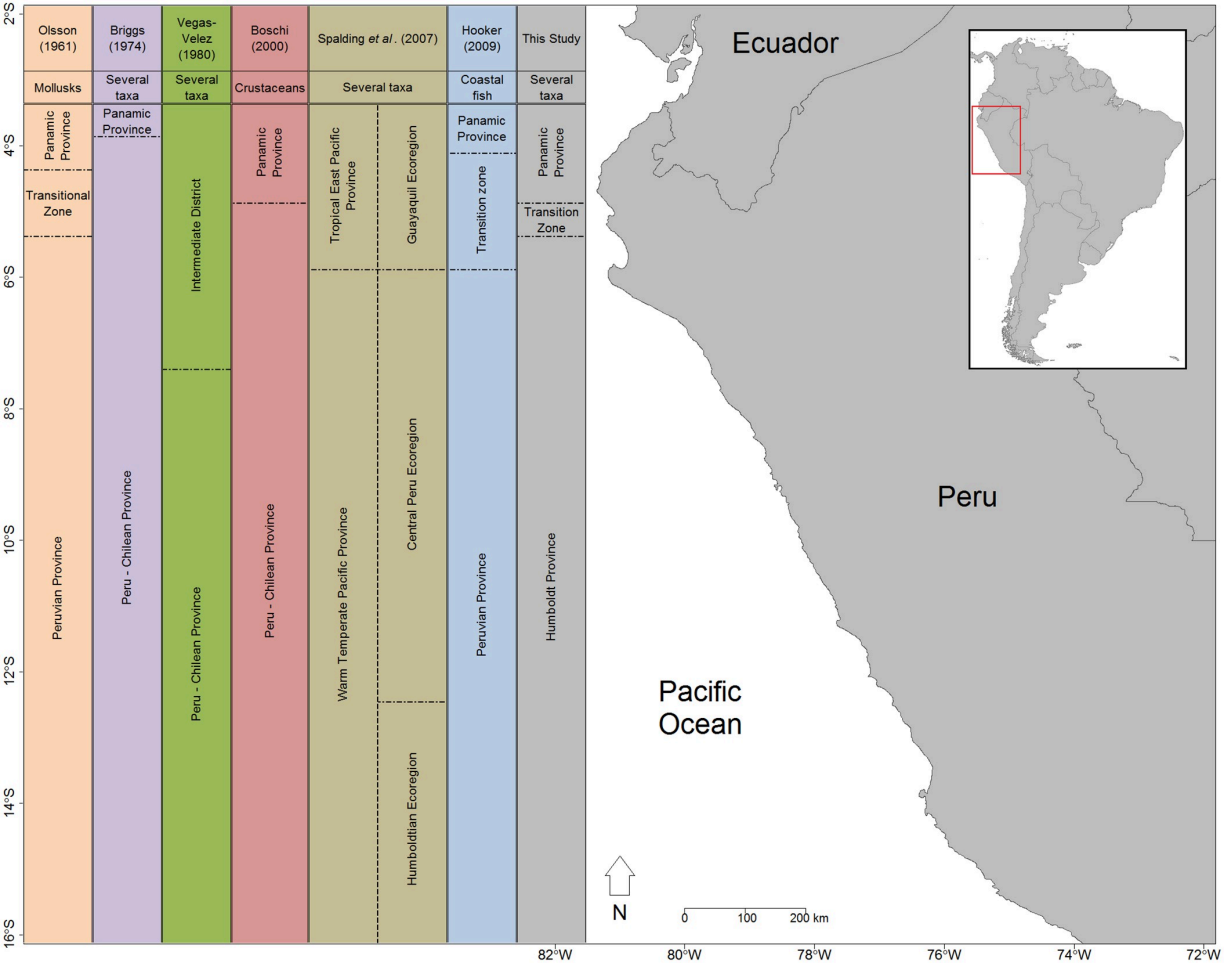
Map of the proposed bioregionalization. Previous bioregionalizations [6, 15, 18, 47, 81] and the zonation revealed in this study.

The study of latitudinal patterns of rocky shore communities throughout the coast of Peru showed the presence of three biogeographic units: a region with affinity to the Panamic province from 3.5 to 4.5° S; a transitional zone between 5 and 5.5° S; and a region from 6 to 13.5° S matching the Humboldt (Peruvian) province (Fig 8). This biogeographical zonation is consistent with previous classifications based on qualitative single-taxa studies [46, 47, 69, 70]. However, the putative limits of the transitional zone differ. Vegas-Vélez [47] proposed an extensive intermediate district from 3.3 to 7° S based on fish and mollusk distributional ranges. Schrödl & Hooker [71] limited the transitional zone from 4 to 6° S after nudibranch surveys, while Tarazona et al. [72] considered a shorter intermediate region between 4 and 5° S. Conversely, Spalding et al. [15] and Costello et al. [73] did not distinguish the transitional unit delimited in our study and the previous reports. Possibly, the expert criteria used by Spalding et al. [15] (see also [51]) may have precluded significative information about transitional areas in this region and elsewhere, as observed in seaweed assemblage changes between Angola and Namibia [74], and in Mediterranean fish assemblages [16]. Regional-scale studies based on records of brachyurans [75], isopods [76], and species endemism [77] suggested that a boundary exists at 5.5° S [73]. Also, Bernard et al. [78] proposed boundaries at 3 and 33° S based on bivalve range limits. Meneses & Santelices [79] suggested reconsidering the biogeographical break for macroalgae from 6 to 12° S due to new records in the area. Such variability in the extent of the transitional zone suggests that border effects may be taxa specific [80]. Community level analysis may reduce those effects by capturing multi taxa variability, resulting in more consistent patterns.

An important factor to consider for understanding biogeographic patterns is the fact that distributional ranges of many species expand throughout the Southeast Pacific coast during El Niño events [48, 81], and some species may persist in refuge areas some years after [35, 82]. This suggests that barriers to dispersal towards higher latitudes may be related to the main equatorward flow of the Humboldt system. Hence, using species records at different years may be problematic in this region, particularly when based on net accumulation of species over time. Moreover, previous studies were conducted using species distribution coming from a wide range of habitats (e.g. soft-bottom, shallow water) without examining the effect of habitat type on biogeographical zonation [46–48, 69, 75]. This study was based only on the variability of rocky shore communities, and thus, the biogeographic zonation proposed is not biased by the variability induced from converging different habitats.

The study region undergoes decadal warm-cold regime shifts [83]. Our work involved a one-time sampling at each site during the cold phase (“La Vieja”) of this cycle, when intensified upwelling extends cold conditions to the northernmost part of the Peruvian coast [40, 83, 84]. This could explain the rather narrow extent of the transitional zone detected in this study in comparison to previous authors [47, 81]. Studies based on data from the 1940’s, mid 1970’s and 1980’s (e.g. [47]) proposed a wider transitional range which may reflect warm decadal conditions (“El Viejo”). Conversely, the transitional zone proposed here only includes sampling sites within 5 to 5.5° S. It could be expected that sites between 6 and 8.5° S should be similar to the transitional area. However, even when the latter latitudes consisted mostly of extensive sandy beaches, and only one rocky shore site was sampled at both 6.5 and 7° S, these communities were more similar to the group of the Humboldt province.

Beta diversity analysis overall indicated significant turnover of taxa community within the transitional zone in comparison to the northern and southern biogeographical units. Significant variations in beta diversity often result from ecological processes, historical events and environmental filtering, resulting in inter-regional differences in species composition [28, 59, 85]. For example, significant changes on species turnover have been reported over strong salinity gradients [86], at oxygen minimum zones boundaries [87] and sea surface temperature gradients throughout biogeographic limits [30, 88, 89]. As such, the multivariate dispersion test used supported the distinction of three biogeographical units along the Peruvian coast. Similarly, Anderson et al. [59] re-analyzed data from Ellingsen & Gray [90], which consisted on samples of soft-bottom invertebrates spanning 15° latitude at the Norwegian continental shelf. Their multivariate dispersion analysis indicated significant differences on beta diversity between southern and northern areas along the latitudinal gradient, therefore suggesting a strong transition from south to north climes.

The variation of relative abundance played an important role in distinguishing biogeographical units, as it has been observed in other regions. At the Northeast Pacific, a subset of species was present throughout the entire latitudinal gradient, but their relative abundances shifted between biogeographical units [20, 22]. In the Southeast Pacific, changes in dominance between macroalgae and mussels along a latitudinal gradient were related to the presence of upwelling centers which tended to be seasonal and weaker towards the south of the examined latitudinal range [52]. Sink et al. [21] reported that the variation in the abundance of conspicuous species such as *Perna perna* (Linnaeus, 1758), *Sargassum elegans* (Suhr, 1840), among others, were paramount for the distinction between subtropical regions at Maputaland and Natal, South Africa. Accordingly, our results suggest that several species may be present throughout the latitudinal gradient, whereas changes in abundance are playing a major role in the delimitation of biogeographic units. For example, the highest percentage of macroalgae abundance was recorded in the Humboldt province.

The environmental information indicated that phosphate concentration, sea surface temperature, photosintetically available radiation and nitrate concentration are the primary variables that interact to delineate the patterns of similarity in community structure along the latitudinal gradient, and henceforth, could be used to predict the biogeographic units. These variables suggest the importance of upwelling shaping the biogeographic structure of the Peruvian province. Several studies stressed that sea surface temperature plays a major role in driving (directly or indirectly), the biogeographic patterns of marine organisms [20, 52, 56]. This occurs in the intertidal via mechanisms like metabolic rate restriction and molecular evolution [91, 92]. Nutrient availability affects intertidal productivity and community structure through bottom-up forcing [93, 94]; and radiation will affect photosynthesis, calcification and body temperature [95–97]. Between upwelling and downwelling areas in South Africa, differences in epilithic microalgae abundance in the intertidal evidenced patterns that were influenced by nutrient concentration [98]. At the Northeast Pacific, Fenberg et al. [56] found that nutrients had a significant influence on biogeographic patterns, possibly associated with variation in upwelling regimes and terrestrial freshwater input. Important upwelling centers in our study region are present at 5, 6, 9 and 12° S, yielding in nutrient-rich, cold and oxygen-poor waters [72, 99]. Consequently, upwelling could be playing an important role in forcing patterns of nutrient, thus influencing community structure. Furthermore, the only latitudinal bin not correctly predicted by the Random Forest model corresponded to the transitional area. Previous studies exploring the relationship between environmental variables and biogeographic structure have also reported a reduced accuracy of the models around biogeographic boundaries [56, 100], attributed to the high environmental variability observed around these areas. Our results corroborated the influence that a suite of oceanographic variables has on similarity patterns of community structure but several other surrogates exist that could be further examined to advance the understanding of their influence on the observed structure.

This study provides the much-needed biogeographical zonation for a poorly studied region in the Southeast Pacific. Changes in sea and land temperatures may be affecting biogeographic patterns in unpredictable ways [20, 41]. In addition, several organisms inhabiting the rocky shores of Peru are harvested by artisanal fishermen [101], which may induce important changes in community structure (e.g. [102]). The impact that such drivers may have on biogeographical zonation remains to be revealed. However, our results increase the precision of broad-scale consensual delimitations and may serve as a baseline for future biogeographic shifts in the face of global change [72, 88, 103]. Marine protected areas in Peru only exist within the Peruvian province [104]. The presence of the other two biogeographic units should be considered in the establishment of further marine protected areas and spatial plans for conservation.

## Supporting information

S1 Table Variables analyzed using Random Forest. (DOC)

S2 Table Complete abundance list of taxa recorded in this study. (XLSX)

S3 Table Complete presence list of taxa recorded in this study. (XLSX)
