# Supplementary material for: Biogeographical zonation of rocky intertidal communities along the coast of Peru (3.5–13.5° S Southeast Pacific)
Source: PLoS One. 2018 Nov 30;13(11):e0208244. doi: 10.1371/journal.pone.0208244 (PMC6267975; doi:10.1371/journal.pone.0208244)
Supplement: S1 Table — (DOC) [file pone.0208244.s001.doc]

**S1 Table.** Variables analyzed using Random Forest (Tyberghein et al. 2012).

| Variable | Original spatial resolution | Temporal range | Source |
| --- | --- | --- | --- |
| Calcite concentration (mol/m^3^) | 5 arcmin (9.2 km) | 2002 - 2009 | Bio-ORACLE |
| Chlorophyll A concentration (max) (mg/m^3^) | 5 arcmin (9.2 km) | 2002 - 2009 | Bio-ORACLE |
| Chlorophyll A concentration (mean) (mg/m^3^) | 5 arcmin (9.2 km) | 2002 - 2009 | Bio-ORACLE |
| Chlorophyll A concentration (min) (mg/m^3^) | 5 arcmin (9.2 km) | 2002 - 2009 | Bio-ORACLE |
| Chlorophyll A concentration (range) (mg/m^3^) | 5 arcmin (9.2 km) | 2002 - 2009 | Bio-ORACLE |
| Cloud fraction (max) (%) | 6 arcmin (11 km) | 2005 - 2010 | Bio-ORACLE |
| Cloud fraction (mean) (%) | 6 arcmin (11 km) | 2005 - 2010 | Bio-ORACLE |
| Cloud fraction (min) (%) | 6 arcmin (11 km) | 2005 - 2010 | Bio-ORACLE |
| Diffuse attenuation coefficiency at 490nm (max) (m^-1^) | 5 arcmin (9.2 km) | 2002 - 2009 | Bio-ORACLE |
| Diffuse attenuation coefficiency at 490nm (mean) (m^-1^) | 5 arcmin (9.2 km) | 2002 - 2009 | Bio-ORACLE |
| Diffuse attenuation coefficiency at 490nm (min) (m^-1^) | 5 arcmin (9.2 km) | 2002 - 2009 | Bio-ORACLE |
| Dissolved oxygen (ml/l) |  | 1898 - 2009 | Bio-ORACLE |
| Nitrate (umol/l) |  | 1928 - 2008 | Bio-ORACLE |
| Photosynthetically Available Radiation (max) (Einstein/m^2^/day) | 5 arcmin (9.2 km) | 1997 - 2009 | Bio-ORACLE |
| Photosynthetically Available Radiation (mean) (Einstein/m^2^/day) | 5 arcmin (9.2 km) | 1997 - 2009 | Bio-ORACLE |
| pH (unitless) |  | 1910 - 2007 | Bio-ORACLE |
| Phosphate |  | 1922 - 1986 | Bio-ORACLE |
| Salinity |  | 1961 - 2009 | Bio-ORACLE |
| Silicate |  | 1930 - 2008 | Bio-ORACLE |
| Sea surface temperature | 1 km | 2002 - 2012 | JPL MUR (https://mur.jpl.nasa.gov/) |

References

1. Tyberghein L, Verbruggen H, Pauly K, Troupin C, Mineur F, De Clerck O. Bio‐ORACLE: a global environmental dataset for marine species distribution modelling. Glob Ecol Biogeogr, 2012; 21(2):272-281.
